# Using Hadamard Transform Multiplexed IR Spectroscopy Together with a Segmented Ion Trap for the Identification of Mobility-Selected Isomers

**DOI:** 10.1021/acs.analchem.3c01340

**Published:** 2023-06-12

**Authors:** Vasyl Yatsyna, Ali H. Abikhodr, Ahmed Ben Faleh, Stephan Warnke, Thomas R. Rizzo

**Affiliations:** Laboratoire de Chimie Physique Moléculaire, École Polytechnique Fédérale de Lausanne, EPFL SB ISIC LCPM, Station 6, CH-1015 Lausanne, Switzerland

## Abstract

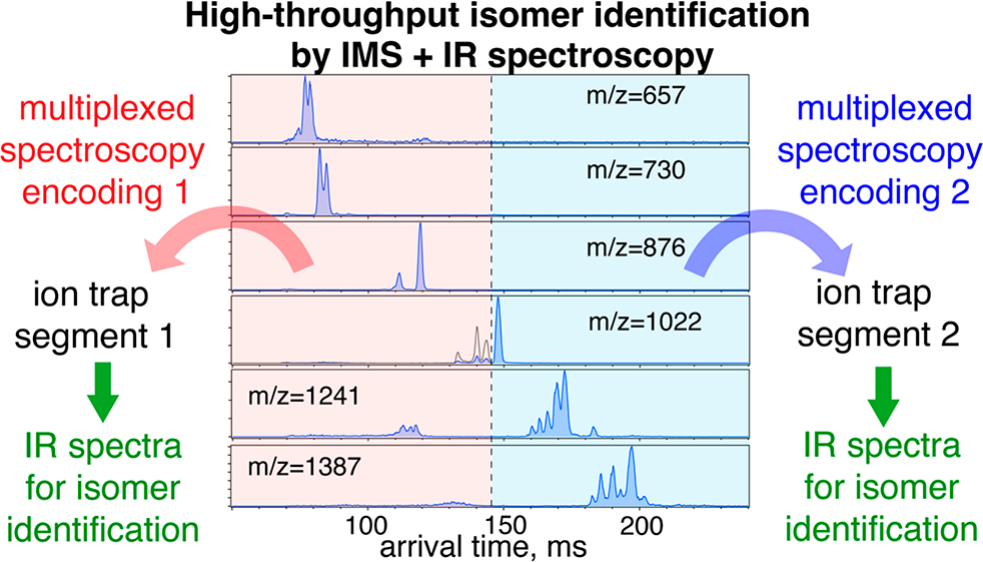

The high isomeric complexity of glycans makes them particularly difficult to analyze. While ultra-high-resolution ion mobility spectrometry (IMS) can offer rapid baseline separation of many glycan isomers, their unambiguous identification remains a challenging task. One approach to solving this problem is to identify mobility-separated isomers by measuring their highly resolved cryogenic vibrational spectra. To be able to apply this approach to complex mixtures at high throughput, we have recently developed a Hadamard transform multiplexed spectroscopic technique that allows measuring vibrational spectra of all species separated in both IMS and mass spectrometry dimensions in a single laser scan. In the current work, we further develop the multiplexing technique using ion traps incorporated directly into the IMS device based on structures for lossless ion manipulations (SLIM). We also show that multiplexed spectroscopy using perfect sequence matrices can outperform standard multiplexing using Simplex matrices. Lastly, we show that we can increase the measurement speed and throughput further by running multiple multiplexing schemes using several SLIM ion traps in combination with simultaneous spectroscopic measurements in the segmented cryogenic ion trap.

## Introduction

High-throughput biomolecular analysis plays a key role in fields such as drug discovery,^1−3^ biomarker screening,^4,5^ glycomics,^6,7^ and metabolomics.^4,8−12^ While the speed and sensitivity of mass spectrometry (MS) makes it well suited for this task, the identification of isomers remains a formidable challenge,^13−18^ particularly in the case of glycans, or oligosaccharides, which display extensive isomeric diversity.^19,20^ To overcome this analytical challenge, MS is often coupled with chromatographic separation techniques and chemical derivatizations that need to be tailored to specific classes of glycans.^21,22^ Even when isomer separation is possible, it remains difficult to identify a specific isomeric form, which hinders understanding the relationship between glycan structures and their biological functions.

Rapid isomer separation has recently become possible with developments in high-resolution ion mobility spectrometry (IMS) such as trapped IMS,^23−25^ cyclic IMS,^26,27^ and structures for lossless ion manipulations (SLIM).^28−33^ Even though IMS separation is not completely orthogonal to MS, continual instrument calibration allows measuring rotationally averaged collision-cross section values, which together with MS/MS spectra can increase the confidence in identifying analytes present in isomeric mixtures. Nevertheless, glycan isomers often show similar MS/MS spectra, which calls for alternative orthogonal identification schemes that are easily coupled to IMS or other high-throughput separation techniques. Cryogenic infrared (IR) ion spectroscopy offers a robust method of fingerprinting glycan isomers while maintaining the high sensitivity of MS detection. We have recently demonstrated that an IR spectrum can be measured relatively quickly (<10 s),^34^ making ion spectroscopy amenable to high-throughput workflows. Moreover, multiplexing spectroscopy with IMS can further increase the throughput.^34,35^ In the current work, we introduce new approaches to multiplexing IR spectroscopy in combination with ultra-high-resolution SLIM-IMS separations. We demonstrate several modifications to our original Hadamard multiplexing technique^35^ and combine them with simultaneous spectroscopic readout in a segmented ion trap. These developments allow us to analyze mixtures with relatively high isomeric complexity, which we demonstrate using oligosaccharide mixtures extracted from a pooled human milk sample. Human milk oligosaccharides (HMOs) play important roles in promoting a healthy gut microbiome and developing the immune system of infants, and they may also provide antimicrobial and antiviral activity.^36−39^ Their high isomeric complexity makes them perfect model systems for the development of high-throughput separation and identification approaches, which can then be extended to other classes of molecules or mixtures with similar levels of complexity.

## Experimental Methods

### Materials

Isomerically pure HMO standards were acquired from Biosynth Ltd. (U.K.) and Dextra Laboratories Ltd. (U.K.). For electrospray, the samples were dissolved in a 50/50 mixture of water/methanol and used without further purification at concentrations of 10–25 μM. A commercial HMO mixture was acquired from Biosynth Ltd. (U.K.) and prepared in a 50/50 mixture of water/methanol and used without further purification at a concentration of ∼10 μM. A pooled human milk sample (0.5 mL, lyophilized) was purchased from Chemie Brunschwig AG (Switzerland), and reconstituted in 1 mL of water. We then performed defatting procedure by centrifuging 200 μL of this sample at 13000 rpm for 30 min at 4 °C. We then mixed the resulting aqueous fraction with ethanol (1:1) and held it at −80 °C for 1 h, which was followed by 30 min of centrifugation at 13000 rpm at 4 °C in order to precipitate the proteins. We then extracted the aqueous fraction containing HMOs and diluted it 60 times before analysis.

### Experimental Apparatus and Spectroscopic Scheme

**Figure 1:**
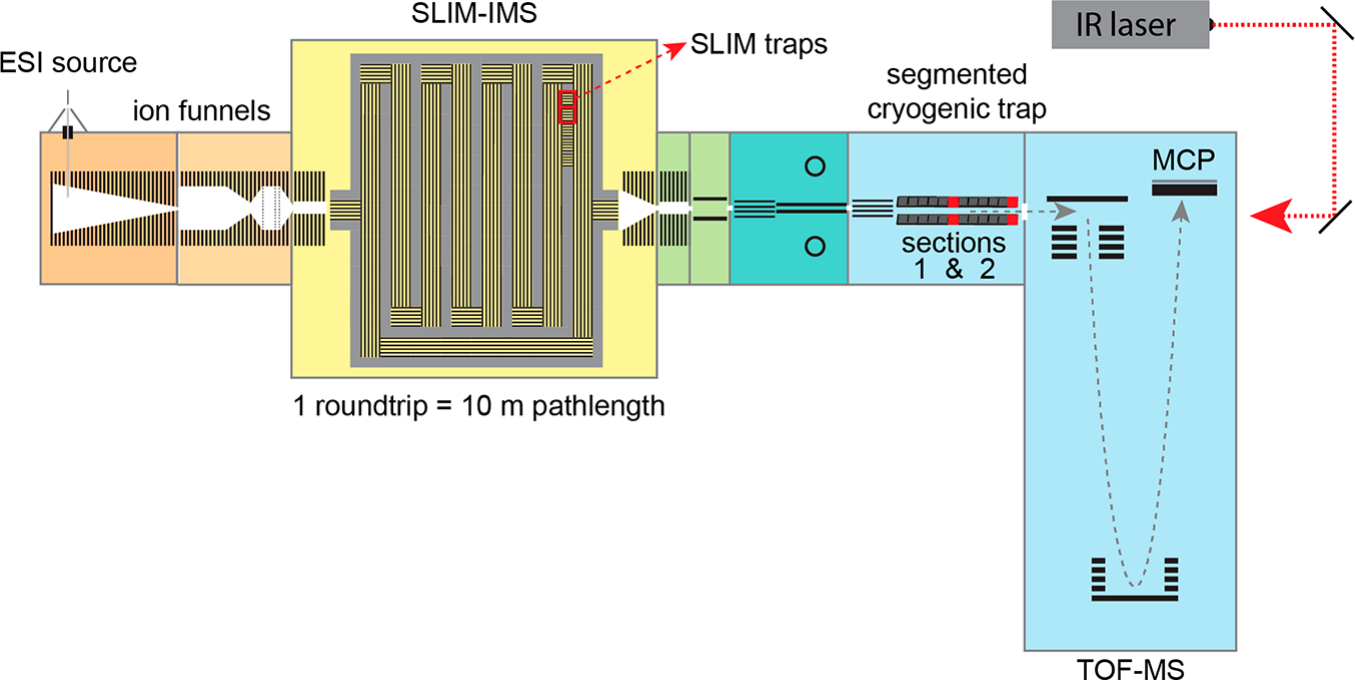
Schematic representation of the experimental setup coupling SLIM-IMS separation and cryogenic ion spectroscopy. Red rectangles show ion traps embedded into SLIM platform that can be used to select and store mobility-selected ions and perform multiplexed spectroscopy. Red blocks show how a segmented cryogenic trap can be split to simultaneously acquire infrared spectra of two separate ion distributions.

The experimental setup used in this work (Figure 1) was described in detail previously.^34^ In brief, analyte ions are generated using nanoelectrospray and transferred into the vacuum chamber through a heated (150 °C) stainless steel capillary. After passing through a set of ion funnels (MassTech, U.S.A.) and a ring-electrode guide, the ions enter an IMS region, where they are separated according to their size, shape, and charge as they drift through 2.2 mbar of nitrogen buffer gas under the influence of electric fields. We use traveling-wave IMS based on SLIM technology, originally developed by Smith and co-workers.^28,30,40^ Our SLIM-IMS module features a 2 m long accumulation region, which is constantly filled from the ion source, and a 10 m long separation region. A relatively short ion packet (0.5–3 ms) is ejected from the accumulation region into the separation region, where it undergoes a single or multiple roundtrips, depending on the required resolving power. A single roundtrip typically results in a resolving power of approximately 200, whereas 10 round trips correspond to a value close to 750.^34^ It is worth noting that our SLIM-IMS module also features on-board ion traps, which can be used for selecting and storing ions as well as for collision-induced dissociation. Fragment ions produced in these traps can subsequently be separated directly by sending them through additional cycles on the SLIM module.

Following SLIM-IMS separation, precursor ions or their fragments are transferred through three differentially pumped regions before entering the cryogenic ion trap. Depending on the mode of operation, ions can either be directly transmitted to a time-of-flight mass spectrometer (TOF-MS; Tofwerk, Switzerland), allowing us to obtain an arrival time distribution (ATD), or they can be trapped for spectroscopic investigation. In the latter case, collisions with cold helium buffer gas cools the ions and allows the formation of weakly bound clusters with N_2_, which is seeded in the buffer gas at a concentration of 10%. The trapped ions are then irradiated with a tunable continuous-wave IR laser (CLT series, IPG, U.S.A.), which can be scanned over the region 3250–3750 cm^–1^. When the laser wavelength is in resonance with a vibrational transition of the messenger-tagged ions, a single-photon is absorbed, leading to the loss of the tag, which is easily detected upon MS. By plotting the fraction of tagged ion signal corresponding to an ion of specific mass and mobility one obtains an IR spectrum, which can be used as a fingerprint for its unambiguous identification.^29,34,41−46^

As a result of cryogenic cooling, the measured IR spectrum reflects the vibrational transitions of the gas-phase molecular ion in its ground state. And since the vibrational structure is an intrinsic property of a molecule, we observe little to no dependence of the measured spectra on the experimental conditions in the ion trap (refer to Figure S1 in the Supporting Information). This characteristic also leads to a high level of day-to-day reproducibility in the measured spectra (Figure S1), making our analytical approach highly suitable for identifying unknown species through database-enabled methods.

It is also worth noting that we typically use SLIM-IMS as a filter, allowing us to select a species with a particular mobility and acquire its spectrum. The design of our segmented cryogenic ion trap^34^ allows multiplexing this process to measure up to 11 species with different ion mobility simultaneously. Since this might still not be enough for the analysis of complex mixtures with high isomeric complexity, we have developed a Hadamard transform multiplexing technique performed after the IMS separation step to further increase the throughput of spectroscopic identification.

### Multiplexed Spectroscopy

Our Hadamard transform multiplexing approach to obtain vibrational spectra of all species separated by IMS has been explained previously.^35^ Here, we give a brief explanation of the main principle of the method. The initial step prior to multiplexing is to measure the IMS-MS profile of the analyzed sample (i.e., the ATD that corresponds to all MS peaks). We then select the window of interest in the arrival time domain and split it into *N* bins. The number and the width of the bins are selected depending on the IMS resolution and the average width of the peaks observed. Hadamard transform multiplexing is then performed at each laser wavelength λ by measuring the encoded matrix **Y**(λ) = **S** × **X**(λ), where **S** is an (*N* × *N*) encoding matrix, **X**(λ) is an (*N* × *k*) matrix that contains the individual mass spectra of *N* bins of interest, and *k* is the length of the MS data vector. The encoding matrix **S** consists of zeros and ones (see Tables S1 and S2 in the Supporting Information), where zeros imply deflecting (discarding) ions in a certain IMS bin, and ones imply transmitting them to the cryogenic trap for IR spectroscopy. Multiplexed measurements of the encoded matrix **Y**(λ) are performed in a row-by-row manner, meaning that the mass spectra of different combinations of separated IMS species that correspond to each row of the **S** matrix are measured. The obtained **Y**(λ) matrix that contains information about various combinations of IMS-MS species can then be demultiplexed, using inverse transformation **X**(λ) = **S**^–1^ × **Y**(λ), in order to obtain the individual MS traces of each IMS bin. This data can then be used to plot the messenger-tagging IR spectra of all IMS-MS peaks of interest. In this work, the encoding process and ion gating were controlled by the digital output module of a PCIe-6320 card (National Instruments) connected to a TTL-driven voltage switch. It is also worth noting that we evaluated the performance of demultiplexing using both inverse transformation **X**(λ) = **S**^–1^ × **Y**(λ) and the maximum likelihood estimation approach^47^ and found that the resulting IR spectra following demultiplexing with these two approaches are almost identical. Nevertheless, we have found an improvement in the quality of the demultiplexed IMS-MS profile using the maximum likelihood estimation approach.

In our previous work,^35^ ions with different mobilities were separated using SLIM-IMS and then transferred to the high-vacuum region of the instrument, where Hadamard transform multiplexing was performed by deflecting the ion packets corresponding to ‘0’ elements of the encoding matrix. The ions that were not deflected were then sent for spectroscopy in the cryogenic trap. For ion trapping, cooling, and messenger-tagging spectroscopy, a short pulse of cooling gas was injected prior to the arrival of ions to the trap. However, for SLIM-IMS separations using extended pathlengths (>10 m), ions present in the isomeric mixtures typically arrive at the cryogenic trap over a broad temporal window (>20 ms), and in that case, multiple gas pulses are required to cool all the separated ions. In order to simplify the multiplexing approach and improve the cooling and messenger-tagging efficiency for all the separated ions, we use an ion trap embedded directly into the SLIM-IMS separation device as an encoding ion gate. In this approach, ions are separated using SLIM-IMS, and then those ion packets that correspond to 1’s of the encoding matrix are diverted into the SLIM trap, whereas other ions simply pass through the SLIM structure and are discarded. The selected, trapped ions are then ejected toward the cryogenic ion trap in the form of a single ion packet. In this way, all the ions are cooled and tagged efficiently, as a single pulse of the cooling gas can be pulsed into the trap at the optimal time, a few milliseconds prior to their arrival. The tagged ions are then irradiated with the IR laser light and analyzed using TOF-MS. Thus, by scanning the IR laser and demultiplexing the encoded TOF-MS data measured at each IR wavelength step, we obtain the messenger-tagging IR spectra of various SLIM-IMS separated species present in the complex mixtures.

Instead of using standard Simplex matrices based on pseudorandom sequences for our Hadamard multiplexed spectroscopy approach, we also explored matrices based on the so-called perfect sequences, also known as two-level autocorrelation sequences,^48^ which may be better suited for multiplexed measurements under shot-noise limited conditions present during the TOF-MS analysis. These sequences (see Table S1) have the lowest number of nonzero elements required to produce improvement in the signal-to-noise (SNR) ratio^48^ but do not significantly increase the shot noise associated with the higher level of signals upon multiplexing compared to nonmultiplexed measurements. Moreover, using multiplexing matrices based on perfect sequences decreases the number of ions stored in the cryogenic trap for spectroscopy, which in turn decreases the chance of overfilling the trap and favorably increases the messenger tagging efficiency, which is important for our spectroscopic scheme.

## Results and Discussion

### Using Perfect Sequences for Multiplexing

**Figure 2:**
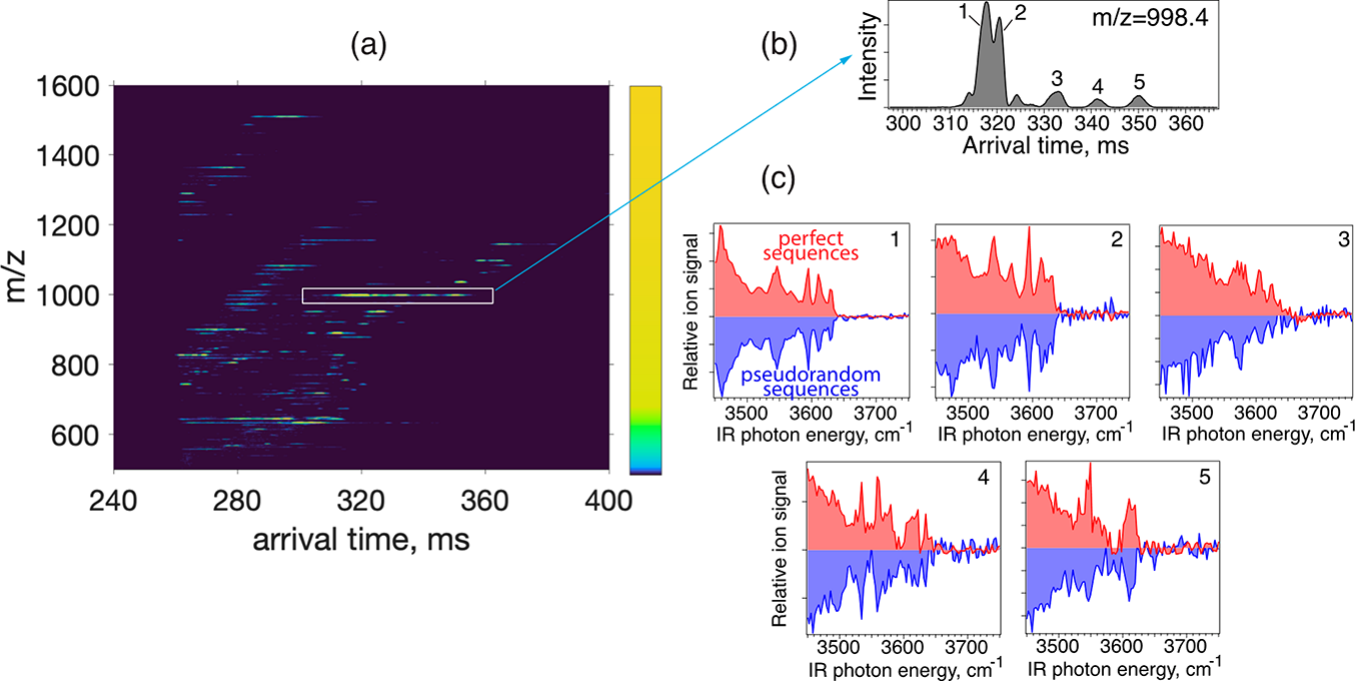
(a) IMS-MS profile (negative ion mode) of the mixture of HMOs extracted from pooled human milk following several separation cycles on our SLIM-IMS platform. (b) Arrival time distribution for *m*/*z* 997.3, which corresponds to the mass of deprotonated isomers of LS-tetrasaccharide. (c) IR spectra of IMS-MS peaks shown in (b) measured in a multiplexed manner, using encoding with pseudorandom sequences (blue) and perfect sequences (red). The SNR improvement due to perfect sequence encoding can be visible.

In order to verify the advantages of multiplexing using perfect sequences as compared to pseudorandom sequences, we applied these approaches to the analysis of HMOs from a pooled human milk sample. For the comparison, we used sequences having the same length, namely, 31 elements, and performed the measurements under the same experimental conditions. We sprayed the mixture of HMOs in negative ion mode and separated them using several cycles on our SLIM-IMS platform (Figure 2a). Figure 2b shows the ATD following two separation cycles for ions with *m*/*z* 997.3. This value corresponds to the mass of deprotonated isomers of LS-tetrasaccharide, and we observe at least six peaks, baseline- or partially separated. In such a case with many peaks present, multiplexing using perfect sequences results in an average SNR increase by a factor of 2.15 compared to standard pseudorandom sequences. Taking into account other HMO species that show a slightly lower number of peaks in their ATDs, we obtain an average SNR increase of 1.6 when using perfect sequences instead of standard pseudorandom ones. This comparison illustrates the benefit in multiplexing ion spectroscopy of complex mixtures using perfect sequences. Nevertheless, it is worth noting that compared to pseudorandom sequences, the number of perfect sequences is limited. For instance, the practical use of multiplexed laser spectroscopy coupled to IMS separation is limited to perfect sequences with lengths of *N* = 7, 13, 21, 31, and 43. In the following, we present the results exclusively using perfect sequences of length 31, and these results further support the effectiveness of the multiplexed spectroscopy based on perfect sequence encoding.

#### High-Throughput Spectroscopy of a Commercial HMO Mixture

**Figure 3:**
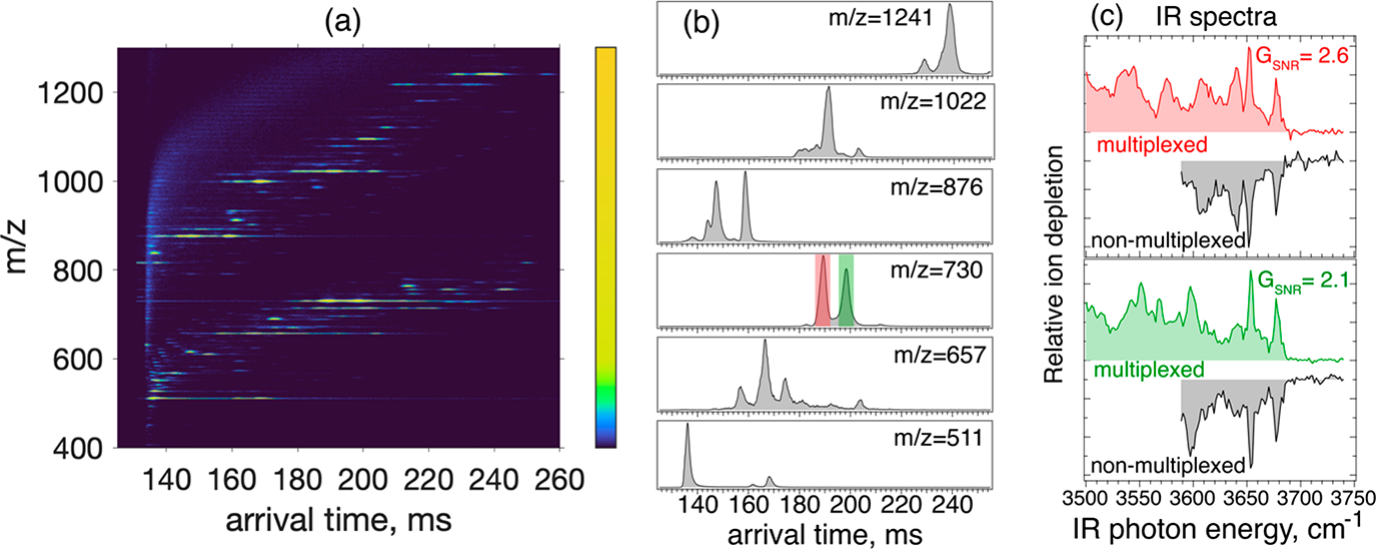
IMS-MS profile (a) and the individual arrival time distributions (b) of the most abundant species in the studied commercial HMO mixture. Single-cycle SLIM-IMS separation corresponding to 10 m drift path length was applied to the ions with *m*/*z* > 800, whereas other ions undergo one additional separation cycle (total path length of 20 m). Panel (c) shows the IR spectra obtained in a multiplexed manner (colored traces) compared to nonmultiplexed measurements (black traces) for the two IMS-MS peaks labeled in panel (b) that correspond to nominal *m*/*z* 730.

Figure 3a shows the IMS-MS profile measured for the commercial HMO mixture (Biosynth Ltd.) following SLIM-IMS separation. The individual ATDs for several mass channels demonstrating isomer heterogeneity are shown in Figure 3b. In order to obtain the spectra of all the separated IMS-MS species in a single laser scan, we applied multiplexed spectroscopy. For this, we split the ATD region between 135 and 255 ms into 31 bins, 3.8 ms wide, covering the majority of species present in the HMO mixture. As mentioned above, compared to our previous work,^35^ here we employ the ion trap on the SLIM device for multiplexing, which simplifies the measurements and increases the efficiency of trapping and cryogenic cooling following separation and multiplexing. Moreover, we have used perfect sequences instead of pseudorandom ones. Multiplexing allowed us to obtain high-quality vibrational spectra of the majority of IMS separated species in a single 18 min laser scan. For comparison, we also performed spectroscopy without multiplexing but under the same experimental conditions. Figure 3c compares the spectra obtained with multiplexing (colored traces) and without multiplexing (black traces) for the two species observed at *m*/*z* 730, which we assign to the reducing-end anomers of LNnT oligosaccharide using our spectroscopic database (Figure S2). We estimate the multiplexing gain in the signal-to-noise ratio for these two species as 2.6 and 2.1 for the first and second ATD peaks, respectively. By comparing the multiplexed and nonmultiplexed data for all other SLIM-IMS separated species, we obtained an average gain in SNR of 2.1, which corresponds to a factor of 4.4 reduction in measurement time. This means that obtaining spectra of similar quality using nonmultiplexed measurements would require ∼79 min rather than 18 min. These results demonstrate the effectiveness of the multiplexed spectroscopy approach in measuring high-quality IR fingerprints of the majority of species present in relatively complex mixtures after high-resolution IMS separation.

#### Combining a Segmented Cryogenic Ion Trap with Hadamard Transform Spectroscopy

In our previous work^34^ we demonstrated that a cryogenic ion trap with multiple reconfigurable trapping sections can be used for multiplexed IR spectral acquisition. For instance, using our current segmented ion trap design with a total length of 110 mm, one can acquire the IR spectra of up to 11 IMS-separated ion packets. In order to increase the throughput of the spectroscopic measurements even further, we can combine simultaneous IR spectral acquisition using the segmented ion trap with Hadamard transform (or perfect sequence) multiplexing.

**Figure 4:**
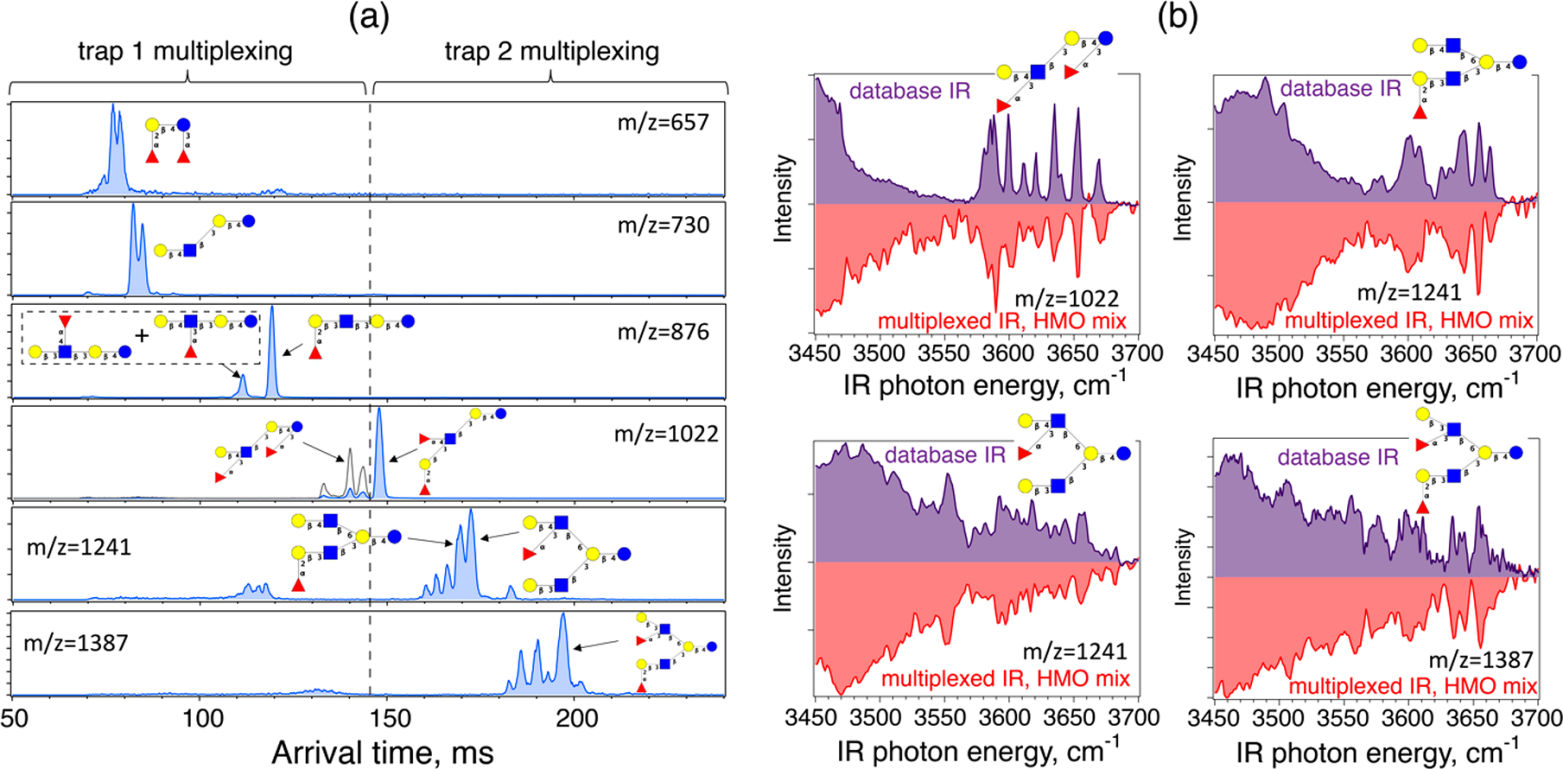
(a) Arrival time distributions of several species found in the pooled human milk HMO extract, together with structures that were unambiguously identified in a single laser scan by performing multiplexed spectroscopy using two cryogenic trap sections. Panel (b) shows the comparison between several messenger-tagging IR spectra obtained in a multiplexed manner and the spectra present in our database, together with the assigned HMO structures.

To demonstrate this approach, we recorded the IR spectra of the mixture of HMOs extracted from pooled human milk using two sections of the segmented cryogenic trap, and for each of the sections we performed multiplexing using perfect sequences. As described above, the multiplexing is performed by modulating the ion gates at the entrances of the ion traps incorporated in the SLIM-IMS device, and in this case, we use two SLIM traps simultaneously. One SLIM ion trap is used for the first half of the ATD (see Figure 4a), and another for the second half of ATD. Multiplexing was implemented using two perfect sequences that were identical, but shifted in time. After ion packet selection according to the perfect sequences, the ions were stored in their respective SLIM ion traps until all other species were transmitted through the SLIM-IMS structure and discarded. The ions stored in these traps were then ejected sequentially and trapped separately in the two sections of the segmented cryogenic trap (see Figure 1), where they were cooled and tagged with N_2_ molecules. All the ions stored in the cryogenic trap were then simultaneously irradiated by the IR laser, and the contents of the two trap sections were sequentially ejected and analyzed separately by the TOF-MS. This allows us to record the two encoded matrices **Y**_1_(λ) and **Y**_2_(λ) containing the information about the two parts of the ATD, and this process is repeated at each laser wavelength step. Following demultiplexing, mass spectra of each IMS bin are obtained, and thus the messenger-tagging IR spectra of the majority of the IMS-MS species are acquired. Figure 4b presents a few examples of the multiplexed IR spectra obtained in this manner, as well as the structures of isomers that are assigned by comparing the measured spectra to our database of isomerically pure standards that we are gradually expanding. Moreover, by comparing the multiplexed and nonmultiplexed spectra acquired under otherwise identical experimental conditions, we estimate a gain in SNR of 1.65, which is an average across various IMS-MS species and corresponds to a factor of 2.7 reduction in measurement time. It is worth noting that compared to multiplexed measurements of the commercial HMO mixture presented above, here we performed a laser scan covering a slightly broader wavenumber range (3450–3700 cm^–1^) and a slightly smaller step size (1.5 cm^–1^), which resulted in a total multiplexed scan duration of ∼28 min. In the future, we plan to employ at least four segments of the cryogenic trap which will reduce the time needed to analyze isomeric mixtures of similar complexity below 15 min. This can be further decreased by optimizing laser stepping routines and tuning ranges to achieve unambiguous identification of isomers present in mixtures with the lowest number of necessary laser steps.

## Conclusions

In this work, we demonstrate high-throughput identification of species present in HMO mixtures with high isomeric complexity through the use of extended-path length SLIM-IMS separations and multiplexed IR spectroscopy. We show how our original method of Hadamard transform multiplexed spectroscopy can be improved by using ion traps embedded into the SLIM-IMS separation device as well as using perfect sequences for encoding instead of pseudorandom sequences. Additionally, we demonstrate efficient coupling of multiplexed spectroscopy with simultaneous spectroscopic readout in the two sections of a segmented cryogenic trap. This not only speeds up the measurements but also greatly increases the range of mobilities that can be analyzed in a single laser scan. We anticipate that further improvements, such as automated demultiplexing and spectral generation, as well as selection of encoding sequence lengths and bin widths based on the IMS-MS profile of the studied mixture, will further simplify the technique for its broad application to a wide variety of complex mixtures.

## Supporting Information Available

Pseudorandom and perfect sequences that can be used for multiplexed spectroscopy; Comparison between the two multiplexed spectroscopy measurements demonstrating consistency of the measured IR spectra; Comparison between the database spectra of LNnT anomers; Measured multiplexed spectra of the commercial HMO mixture (PDF)
