# Supplementary material for: Using Hadamard Transform Multiplexed IR Spectroscopy Together with a Segmented Ion Trap for the Identification of Mobility-Selected Isomers
Source: Anal Chem. 2023 Jun 12;95(25):9623–9. doi: 10.1021/acs.analchem.3c01340 (PMC10308330; doi:10.1021/acs.analchem.3c01340)
Supplement: Supplementary file 1 — ac3c01340_si_001.pdf [file ac3c01340_si_001.pdf]

Supporting Information

Using Hadamard transform multiplexed IR spectroscopy together with a segmented ion trap for the identification of mobility-selected isomers

Vasyl Yatsyna, Ali H. Abikhodr, Ahmed Ben Faleh, Stephan Warnke, and Thomas R. Rizzo*

Laboratoire de Chimie Physique Moléculaire, École Polytechnique Fédérale de Lausanne, EPFL SB ISIC LCPM, Station 6, CH-1015 Lausanne, Switzerland

*email: thomas.rizzo@epfl.ch

Table of Content:

Table S1. List of perfect sequences, also known as two-level autocorrelation sequences,[1] which can be used to construct encoding matrices for multiplexed spectroscopy. N corresponds to sequence length, and z equals to the number of non-zero elements. S2

Table S2. List of pseudorandom sequences[2] that are used to construct Simplex matrices for Hadamard transform multiplexing. N corresponds to sequence length, and z equals to the number of non-zero elements. S2

Figure S1. Comparison between the two multiplexed spectroscopy measurements performed on 02.09.2022 and 07.09.2022 for HMO mixture extracted from pooled human milk. Panel (a) compares arrival time distributions for the HMO observed at m/z=997.3 ([M-H]$^-$), and panels (b)-(f) compare the measured IR spectra that correspond to the observed IMS peaks 1-5. The measurements were conducted under slightly different electric field conditions in the cryogenic ion trap, resulting in slight changes in the messenger-tagging efficiency and signal-to-noise ratio. However, these variations have minimal impact on the peak positions and relative intensities, which are crucial for species identification. S3

Figure S2. The database messenger-tagging IR spectra of LNnT anomers (blue and purple traces) compared with multiplexed IR spectra of the commercial HMO mixture, obtained for IMS peak 1 (red, top) and IMS peak 2 (green, bottom)....S3

References....S4

Table S1. List of perfect sequences, also known as two-level autocorrelation sequences,[1] which can be used to construct encoding matrices for multiplexed spectroscopy. N corresponds to sequence length, and z equals to the number of non-zero elements.

N	z	Sequence
7	3	1 1 0 0 0 1 0
13	4	1 1 0 1 0 0 0 0 0 1 0 0 0
21	5	1 1 0 0 1 0 0 0 0 0 0 0 0 0 1 0 1 0 0 0 0
31	6	1 1 0 1 0 0 0 0 1 0 0 0 1 0 0 0 0 0 1 0 0 0 0 0 0 0 0 0 0 0 0
43	7	1 1 0 1 0 0 0 1 0 0 0 0 1 0 0 0 0 0 0 0 1 0 0 0 0 0 0 0 0 0 1 0 0 0 0 0 0 0 0 0 0 0 0

Table S2. List of pseudorandom sequences[2] that are used to construct Simplex matrices for Hadamard transform multiplexing. N corresponds to sequence length, and z equals to the number of non-zero elements.

N	z	Sequence
7	4	1 1 1 0 1 0 0
11	6	1 1 0 1 1 1 0 0 0 1 0
15	8	0 0 0 1 0 0 1 1 0 1 0 1 1 1 1
19	10	1 1 0 0 1 1 1 1 0 1 0 1 0 0 0 0 1 1 0
23	12	1 1 1 1 1 0 1 0 1 1 0 0 1 1 0 0 1 0 1 0 0 0 0
31	16	0 0 0 0 1 0 0 1 0 1 1 0 0 1 1 1 1 1 0 0 0 1 1 0 1 1 1 0 1 0 1
35	18	0 0 1 0 0 1 1 0 1 0 1 0 0 0 0 1 0 0 1 1 1 0 1 1 1 1 1 0 0 0 1 1 1 0 1
43	22	1 1 0 0 1 0 1 0 0 1 1 1 0 1 1 1 1 1 0 0 0 1 0 1 1 1 0 0 0 0 0 1 0 0 0 1 1 0 1 0 1 1 0
47	24	1 1 1 1 1 0 1 1 1 1 0 0 1 0 1 0 1 1 1 0 0 1 0 0 1 1 0 1 1 0 0 0 1 0 1 0 1 1 0 0 0 0 1 0 0 0 0

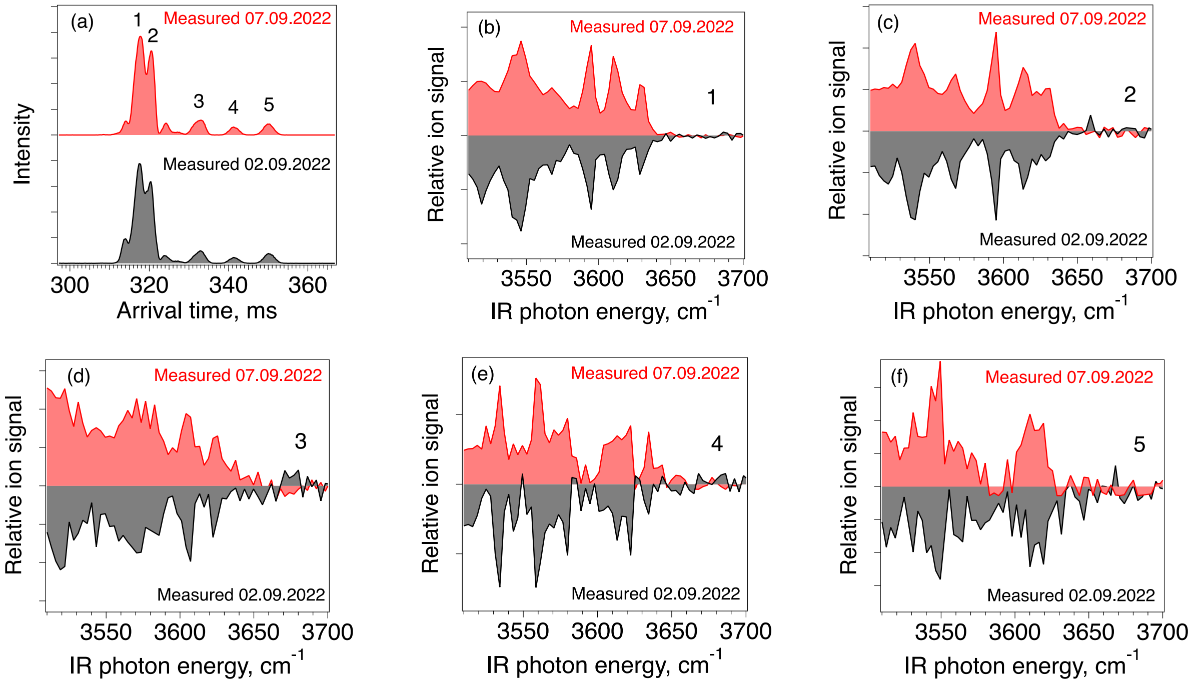

Figure S1. Comparison between the two multiplexed spectroscopy measurements performed on 02.09.2022 and 07.09.2022 for HMO mixture extracted from pooled human milk. Panel (a) compares arrival time distributions for the HMO observed at m/z=997.3 ([M-H]$^-$), and panels (b)-(f) compare the measured IR spectra that correspond to the observed IMS peaks 1-5. The measurements were conducted under slightly different electric field conditions in the cryogenic ion trap, resulting in slight changes in the messenger-tagging efficiency and signal-to-noise ratio. However, these variations have minimal impact on the peak positions and relative intensities, which are crucial for species identification.

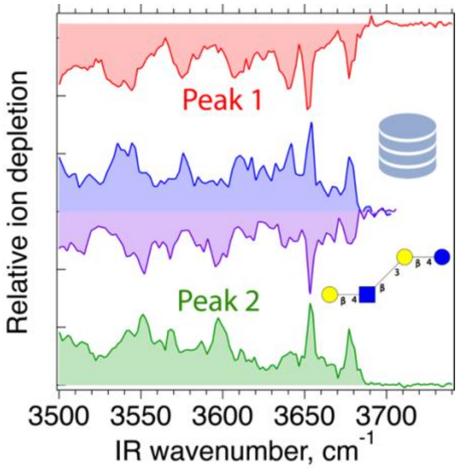

Figure S2. The database messenger-tagging IR spectra of LNnT anomers (blue and purple traces) compared with multiplexed IR spectra of the commercial HMO mixture, obtained for IMS peak 1 (red, top) and IMS peak 2 (green, bottom).

References

(1) Wuttig, A. Optimal transformations for optical multiplex measurements in the presence of photon noise. *Appl. Opt.* **2005**, *44*, 2710-2719.

(2) Harwit, M.; Sloane, N. J. A. In *Hadamard Transform Optics*, Harwit, M.; Sloane, N. J. A., Eds.; Academic Press, 1979, pp 1-19.
